# Low-Temperature-Induced Controllable Transversal Shell Growth of NaLnF_4_ Nanocrystals

**DOI:** 10.3390/nano11030654

**Published:** 2021-03-08

**Authors:** Deming Liu, Yan Jin, Xiaotong Dong, Lei Liu, Dayong Jin, John A. Capobianco, Dezhen Shen

**Affiliations:** 1 State Key Laboratory of Luminescence and Applications, Changchun Institute of Optics Fine Mechanics and Physics Chinese Academy of Sciences, Changchun 130033, China; jinyan@mails.ucas.edu.cn (Y.J.); dpldyx@126.com (X.D.); liulei@ciomp.ac.cn (L.L.); 2 Center of Materials Science and Optoelectronics Engineering, University of Chinese Academy of Sciences, Beijing 100049, China; 3 School of Chemistry and Chemical Engineering, Guizhou University, Guiyang 550025, China; 4 Institute for Biomedical Materials and Devices, Faculty of Science, University of Technology Sydney, Sydney, NSW 2007, Australia; 5 Department of Chemistry and Biochemistry and Center for NanoScience Research, Concordia University, Montreal, QC H4B 1R6, Canada

**Keywords:** NaLnF_4_, core–shell, UCNP, nanocrystal engineering

## Abstract

Highly controllable anisotropic shell growth is essential for further engineering the function and properties of lanthanide-doped luminescence nanocrystals, especially in some of the advanced applications such as multi-mode bioimaging, security coding and three-dimensional (3D) display. However, the understanding of the transversal shell growth mechanism is still limited today, because the shell growth direction is impacted by multiple complex factors, such as the anisotropy of surface ligand-binding energy, anisotropic core–shell lattice mismatch, the size of cores and varied shell crystalline stability. Herein, we report a highly controlled transversal shell growth method for hexagonal sodium rare-earth tetrafluoride (β-NaLnF_4_) nanocrystals. Exploiting the relationship between reaction temperature and shell growth direction, we found that the shell growth direction could be tuned from longitudinal to transversal by decreasing the reaction temperature from 310 °C to 280 °C. In addition to the reaction temperature, we also discussed the roles of other factors in the transversal shell growth of nanocrystals. A suitable core size and a relative lower shell precursor concentration could promote transversal shell growth, although different shell hosts played a minor role in changing the shell growth direction.

## 1. Introduction

Lanthanide-doped core–shell upconverting nanoparticles (UCNPs) have been widely studied due to their advantages of enhancing luminescence, improving emission stability, separating multiple dopants, etc., which promises many specific applications, such as bioimaging [1,2,3,4,5,6], drug delivery [7,8,9], biosensors [10,11,12], 3D display [13], optical multiplexing [14,15], photovoltaics [16] and photodynamic therapy [17]. Precisely controlling the shell growth direction is essential in order to guarantee the desired structure and avoid the unwanted crosstalk between different emitters in the multifunctional core–shell nanocrystals [18]. 

Hexagonal phase NaLnF_4_ nanocrystals, as excellent luminescence host materials, have similar unit cell parameters in a hexagonal crystallographic structure. They are usually covered by two groups of planes: {001} planes and {100} planes, presented by two equivalent vertical faces and the six equivalent lateral faces. The differences in atom arrangement between the two kinds of planes make it feasible to control the anisotropic shell growth. 

Due to many efforts in the synthesis of NaLnF_4_ core–shell nanocrystals, some high-quality core–shell or heterogeneous nanocrystals with different shapes have been successfully synthesized [19,20,21,22,23,24,25]. Among them, the isotropic and longitudinal shell growth of NaLnF**_4_** core–shell nanocrystals have been reported more often. However, there are few reports of the transversal shell growth of NaLnF_4_ nanocrystals. Although Yan’s group successfully fabricated disk-like core–shell NaLnF_4_ nanocrystals [16], they were more focused on studying the optical properties of the nanomaterials, rather than the mechanism of the transversal shell growth control. The mechanism of the transversal shell growth control is not yet clear. For example, Lee’s group explained that the transversal shell growth of NaYF_4_ nanocrystals was attributable to the preference of oleic acid to attach on (001) planes [26], while in another report [27], the longitudinal shell growth of NaYF_4_ was explained by oleic acid preferring to attach on (100) planes. Obviously, oleic acid cannot prefer to attach to (001) and (100) planes at the same time. In our previous report [28], we found that oleic acid prefers to coat on (001) planes while the oleate anions prefer to coat on (100) planes. A lower molar ratio of oleate anions to oleic acid (OA**^−^**/OAH) would promote the transversal shell growth. According to density functional theory (DFT) simulation results, the binding energy of oleates on each facet is much stronger than that of oleic acid on each facet. The (001) planes usually receive much weaker surfactant coating than (100) planes, which makes the controllable transversal shell growth challenging. Therefore, a deep and systematic study of the transversal shell growth mechanism is required to explore the reason behind the anisotropic shell growth.

In this work, a method for the controllable synthesis of two-dimensional (2D) core–shell NaLnF_4_ nanocrystals is presented. The synthesis was achieved by controlling the transversal shell growth with a relatively lower reaction temperature. We found that in the condition of a low oleate/oleic acid ratio, the reaction temperature was the major factor for tuning the shell growth direction. Varied reaction temperature would change the surfactant binding status on certain facets of the nanocrystals. Transversal shell growth, isotropic shell growth and longitudinal shell growth of NaLnF_4_ nanocrystals were respectively achieved at a relatively lower temperature (~280 °C), an intermediate temperature (~300 °C), and a relatively higher temperature (~310 °C). In addition, other relevant reaction factors for controllable transversal shell growth (e.g., core size, lattice mismatch and shell precursor concentration) were studied carefully as well.

## 2. Materials and Methods

The following reagents were used as received without further purification: thulium chloride hexahydrate (TmCl_3_·6H_2_O, 99.99%), yttrium chloride hexahydrate (YCl_3_·6H_2_O, 99.99%), ytterbium chloride hexahydrate (YbCl_3_·6H_2_O, 99.998%), gadolinium chloride hexahydrate (GdCl_3_·6H_2_O, 99.9%), sodium trifluoroacetate (CF_3_COONa, 98%), sodium hydroxide (NaOH, 98%), ammonium fluoride (NH_4_F, 99.99%), oleic acid (OA, 90%), 1-octadecene (ODE, 90%).

### 2.1. Synthesis of 7.5 nm β-NaGdF_4_ Cores

The β-NaGdF_4_ core nanocrystals were synthesized by a modified reported method. In a typical procedure, 1 mL of GdCl_3_ in methanol (1.0 mmol) was mixed with OA (6 mL) and ODE (15 mL) in a 100 mL three-neck round-bottom flask. The mixture was degassed under Ar flow during heating up to 150 °C, followed by a 30 min isothermal reaction to form a clear solution, and then cooled down to room temperature. Ten milliliters of a methanol solution containing NH_4_F (4 mmol) and NaOH (2.5 mmol) was added to the flask and then stirred for 60 min. The solution was slowly heated up to 110 °C and kept at 110 °C for 30 min to completely remove the methanol and any residual water. Then the reaction solution was quickly heated up to 250 °C and kept isothermally for 1.5 h, before being cooled down to room temperature. Ethanol was added to precipitate the nanocrystals, which were transferred to two 50 mL centrifuge tubes and centrifuge-washed four times with cyclohexane, ethanol and methanol (volume ratio = 2:3:2). The obtained nanocrystals were re-dispersed in 10 mL of cyclohexane (20.5 mg/mL).

### 2.2. Synthesis of 26 nm β-NaYF_4_ Cores

The β-NaYF_4_ core nanocrystals were synthesized by a modified reported method. In a typical procedure, a methanol solution of (2 mL) of YCl_3_ (1 mmol) was mixed with OA (6 mL) and ODE (15 mL) in a 100 mL three-neck round-bottom flask. The mixture was degassed under Ar flow during the heating up to 150 °C followed by a 30 min isothermal reaction to form a clear solution, and then cooled down to room temperature. Ten milliliters of methanol containing NH_4_F (4 mmol) and NaOH (2.5 mmol), was added into the flask and then stirred for 60 min. The solution was slowly heated up to 110 °C and kept at 110 °C for 30 min to completely remove the methanol and any residual water. Then the reaction solution was quickly heated up to 300 °C and kept isothermally for 1.5 h, before being cooled down to room temperature. Ethanol was added to precipitate the nanocrystals, which were transferred to two 50 mL centrifuge tubes and centrifuge-washed four times with cyclohexane, ethanol and methanol (volume ratio = 2:3:2). The obtained nanocrystals were re-dispersed in 10 mL of cyclohexane with a concentration of 18.5 mg/mL. 

### 2.3. Temperature Effect on the Shell Growth of NaYF_4_ Nanocrystals

A methanol solution (1 mL) of YCl_3_ (0.2 mmol) was mixed with OA (6 mL) and ODE (6 mL) in a 100 mL three-neck round-bottom flask. The mixture solution was degassed under Ar flow during the heating up to 160 °C followed by a 30 min isothermal reaction to form a clear solution, and then cooled down to 100 °C. One milliliter of 26 nm NaYF_4_ nanocrystals in cyclohexane stock solution (0.1 mmol) was added into the flask, followed by the addition of 0.25 mmol sodium trifluoroacetate. The solution was slowly heated up to 120 °C and kept at 120 °C for 30 min to completely remove the cyclohexane and any residual water. Then, the reaction solution was quickly heated up to 280 °C or 300 °C or 310 °C and kept isothermally for 1.5 h, before being cooled down to room temperature. Ethanol was added to precipitate the nanocrystals, which were transferred to two 50 mL volume centrifuge tubes and centrifuge-washed four times with cyclohexane, ethanol and methanol (volume ratio = 2:3:2). The obtained nanocrystals were re-dispersed in 3 mL of cyclohexane. 

### 2.4. Synthesis of 16 nm × 21 nm NaGdF_4_/NaYF_4_ Nanocrystals

A methanol solution (4 mL) of YCl_3_ (0.8 mmol) was mixed with 9 mL OA and 9 mL ODE in a 50 mL three-neck round-bottom flask. The mixture solution was degassed under Ar flow during the heating up to 160 °C followed by a 30 min isothermal reaction to form a clear solution, and then cooled down to 100 °C. Then, 0.1 mmol 7.5 nm NaGdF_4_ nanocrystals in cyclohexane stock solution (1 mL) was added into the flask, followed by the addition of 2 mmol sodium trifluoroacetate. The solution was slowly heated up to 150 °C and kept at 150 °C for 20 min to completely remove the cyclohexane and any residual water. Then, the reaction solution was quickly heated up to 280 °C and kept isothermally for 1.5 h, before being cooled down to room temperature. Ethanol was added to precipitate the nanocrystals, which were transferred to two 50 mL volume centrifuge tubes and centrifuge-washed four times with cyclohexane, ethanol and methanol (volume ratio = 2:3:2). The obtained nanocrystals were re-dispersed in 5 mL of cyclohexane (21 mg/mL).

### 2.5. Synthesis of 25.5 nm NaGdF_4_/NaYF_4_ Nanocrystals

A solution of 0.4 mmol YCl_3_ in methanol was mixed with 6 mL OA and 6 mL ODE in a 50 mL three-neck round-bottom flask. The mixture solution was degassed under Ar flow during the heating up to 160 °C followed by a 30 min isothermal reaction to form a clear solution, and then cooled down to 100 °C. Then one milliliter 16 nm × 21 nm NaGdF_4_/NaYF_4_ nanocrystals (21 mg/mL) in cyclohexane stock solution was added into the flask, followed by the addition of 1 mmol of sodium trifluoroacetate. The solution was slowly heated up to 150 °C and kept at 150 °C for 20 min to completely remove the cyclohexane and any residual water. Then the reaction solution was quickly heated up to 300 °C and kept isothermally for 1.5 h, before being cooled down to room temperature. Ethanol was added to precipitate the nanocrystals, which were transferred to two 50 mL centrifuge tubes and centrifuge-washed four times with cyclohexane, ethanol and methanol (volume ratio = 2:3:2). The obtained nanocrystals were re-dispersed in 6 mL of cyclohexane (19 mg/mL).

### 2.6. Shell Precursor Concentration Effect to Transversal Shell Growth

OA-ODE solutions of 0.2 mmol YCl_3_ and 0.5 mmol sodium trifluoroacetate were used for the low shell precursor concentration condition; 0.4 mmol YCl_3_, 2 mmol sodium trifluoroacetate were used for the high shell precursor concentration. The other condition was kept the same.

A certain amount of YCl_3_ was mixed with 6 mL OA and 6 mL ODE in a 50 mL three-neck round-bottom flask. The mixture solution was degassed under Ar flow during the heating up to 160 °C followed by a 30 min isothermal reaction to form a clear solution, and then cooled down to 100 °C. One milliliter 16 nm × 21 nm NaGdF_4_/NaYF_4_ nanocrystals (21 mg/mL) in cyclohexane stock solution was added into the flask, followed by the addition of a certain amount of sodium trifluoroacetate. The solution was slowly heated up to 150 °C and kept at 150 °C for 20 min to completely remove the cyclohexane and any residual water. Then the reaction solution was quickly heated up to 280 °C and kept isothermally for 1.5 h, before being cooled down to room temperature. Ethanol was added to precipitate the nanocrystals, which were washed four times with cyclohexane, ethanol and methanol. 

### 2.7. Preparing Shell Precursor for the Transversal Shell Growth

A solution of 2 mmol LnCl_3_ (GdCl_3_ or YCl_3_ or YbCl_3_) was mixed with 10 mL OA and 10 mL ODE in a 50 mL three-neck round-bottom flask. The mixture solution was degassed under Ar flow during the heating up to 160 °C followed by a 45 min isothermal reaction to form a clear solution, followed by the addition of 5 mmol of sodium trifluoroacetate and stirring for another 30 min and then cooling down to room temperature. 

### 2.8. Transversal Shell Growth for NaGdF_4_/NaLnF_4_ (NaYF_4_ or NaYbF_4_) Nano-Disks

In a typical protocol, 1 mL of stored solution of 25.5 nm NaGdF_4_/NaYF_4_ nanocrystals (19 mg/mL) was mixed with 6 mL OA and 6 mL ODE in a 50 mL three-neck round-bottom flask. The mixture solution was degassed under Ar flow during the heating to 280 °C and the shell precursor was injected with a speed of 0.1 mL (0.02 mmol NaLnF_4_ shell precursor) per 5 min, repeating injection until the required amount of shell precursor was reached. After the last injection, the reaction temperature was kept at 280 °C for 20 min, then the reaction solution was cooled to room temperature. Centrifuge precipitation of the product was performed at 4000 rpm for 10 min, then the precipitate was washed with cyclohexane, ethanol and methanol three times.

### 2.9. TEM Characterization

Both standard TEM and high-resolution TEM were performed on the same sample using a 200 keV JEOL JEM-2100F microscope. Powder X-ray diffraction (XRD) patterns were obtained on a PANalytical X’Pert Pro MPD X-ray diffractometer using Cu K*α*1 radiation (40 kV, 40 mA, l = 0.15418 nm). The XRD samples were prepared by repeatedly drying drops of nanocrystal dispersions in cyclohexane cast on a zero background silicon wafer. The X-ray photoelectron spectroscopy (XPS) was characterized by Thermo Scientific, ESCALAB250Xi. X-ray source: mono-chromated Al K alpha (energy 1486.68 eV); power: 150 W (13 kV × 12 mA).

## 3. Results

**Figure 1:**
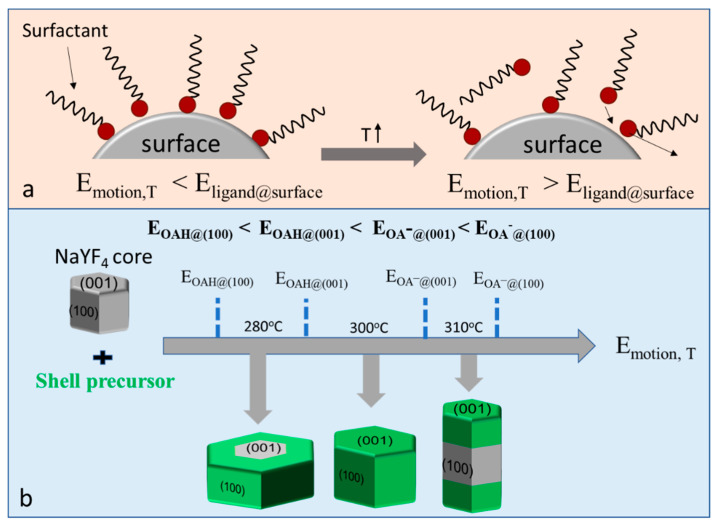
(**a**) Scheme of the relationship between the reaction temperature and the passivation status of surfactant ligands on the crystal surface: the thermal motion energy of the surfactant molecule (*E_motion,T_*) increases as the temperature rises. When *E_motion,T_* > *E_ligand@surface_* (surfactant binding energy on the crystal surface), the surfactant will have an increased chance of being shaken off from the surface. (**b**) Scheme of tuning the shell growth direction for hexagonal NaYF_4_ nanocrystal by adjusting the reaction temperature.

As shown in Figure 1a, we modeled the coating status of surface ligands on the crystal surface, well coated and partially released. The coating status of surfactant ligand was determined by two factors: the binding energy between surfactant and crystal surface, *E_ligand@surface_*, which is decided by the features of the surfactants and planes; and the thermal motion energy of ligands, *E_motion,T_*, which is mainly determined by reaction temperature. The facet passivation status could be “on” (surfactants well attached) or “off” (surfactants partially shaken off), which is decided by the relative value of surfactant binding energy, *E_ligand@surface_*, and the thermal motion energy of surface ligands, *E_motion,T_*. A higher value of *E_motion,T_* will give surfactant ligand a higher frequency to be shaken off from the surface. Therefore, selecting a suitable value of *E_motion,T_*, which will make one plane have more of a chance to become “off” compared to other planes, will induce the shell preferentially grow along this direction. According to our previous work [28], the binding energy values of OAH and OA^−^ at different planes, (100) and (001), follow the order: *E_OAH@(100)_* < *E_OAH@(00_*_1)_ < *E_OA_^−^_@(001)_* < *E_OA_^−^_@(100)_*. Therefore, by adjusting the reaction temperature it should be possible to tune the shell growth direction of NaLnF_4_ nanocrystals (Figure 1b). For example, at a relatively lower temperature, *E_OAH@(100)_* < *E_motion,T_* < *E_OAH@(001_*_)_ < *E_OA_^−^_@(001)_* < *E_OA_^−^_@(100)_*, only the (100) plane will be less passivated by the surfactant due to OAH being shaken off from the (100) plane, which would result in transversal shell growth. When the temperature was increased to a suitable value, resulting in *E_OAH@(100)_* < *E_OAH@(001)_* < *E_motion,T_* < *E_OA_^−^_@(001)_* < *E_OA_^−^_@(100)_*, the passivation on both (001) and (100) planes would be released and isotropic shell growth would occur. Upon further increasing temperature to make *E_OAH@(100)_* < *E_OAH@(001_*_)_ < *E_OA_^−^_@(001)_* < *E_motion,T_* < *E_OA_^−^_@(100)_*, only the (100) plane would be well passivated by oleates, which would result in longitudinal shell growth.

**Figure 2:**
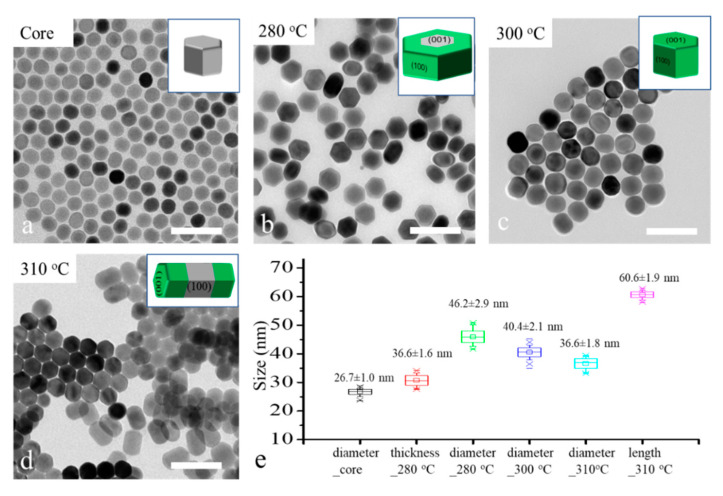
TEM images of core-only (**a**) and core–shell nanocrystals, synthesized at a different temperature, 280 °C (**b**), 300 °C (**c**) and 310 °C (**d**), and their statistical graph of size of core and core–shell nanocrystals. (**e**) All scale bar is 100 nm.

To reveal the role of reaction temperature for the shell growth direction control, 26 nm -NaYF_4_ core nanocrystals were applied as the core for shell growth under different temperatures, 280 °C, 300 °C and 310 °C, in a heating-up method. The TEM images and size distribution graph of the core and core–shell nanocrystals are shown in Figure 2. We can see the major morphology changes as the temperature increased from 280 °C to 310 °C. The disk-like nanocrystals (Figure 2b) suggest a transversal shell growth occurred at 280 °C; the spherical nanocrystals (Figure 2c) illustrate that an isotropic shell growth occurred at 300 °C; and the rod-like nanocrystals (Figure 2d) reveal that a longitudinal shell growth occurred at 310 °C. These results are coherent with our former hypothesis. The size distribution of core and core–shell in Figure 2e shows that the thickness of nano-disk core–shell nanocrystal (36.6 nm) was slightly increased compared with the diameter of the core nanocrystals (26.7 nm), although the major shell growth direction was transversal. To achieve highly controllable transversal shell growth of NaLnF_4_ nanocrystals, the effect of other relative factors such as core size, shell host and shell precursor injection rate need to be considered.

**Figure 3:**
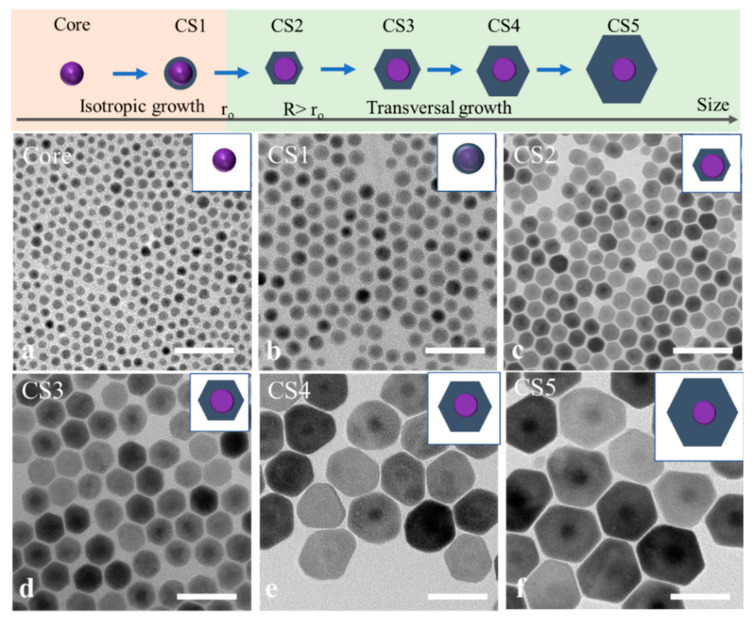
TEM images of core-only NaGdF_4_:Yb^3+^, Tm^3+^ nanoparticles (NPs) (**a**) and NaGdF_4_:Yb, Tm@NaYF_4_ core–shell NPs with different amounts of shell precursors ((**a**) 1 mL, (**b**) 2 mL, (**c**) 4 mL, (**d**) 8 mL, (**e**) 16 mL, (**f**) 32 mL). The 2D shell growth process with increasing precursor injection under 280 °C, scale bar 50 nm.

To study the effect of the core size to the transversal shell growth, 7.5 nm NaGdF_4_ nanocrystals as the cores were applied for the continuous growth of shells with different thickness at 280 °C. Differently sized NaGdF_4_/NaYF_4_ core–shell nanocrystals were collected as the shell precursors were continuously injected into the reaction system. The crystal phase of the core and core–shell nanocrystals remained in the hexagonal phase, as indicated by the X-ray diffractograms shown in Figure S1. The surface information of the core and core–shell structure was characterized by XPS, as shown in Figure S2, which shows the Gd4d and Y3d peaks. The morphology changes of core and core–shell nanocrystal with increased sizes are recorded in TEM images shown in Figure 3. Interestingly, the core did not follow the transversal shell growth at the beginning but grew isotopically (Figure 3b). With more shell growth, 13 nm spherical nanocrystals were transferred into 16 nm hexagonal prism core–shell nanocrystals (Figure 3c). Beyond this size, the transversal shell growth trend gradually formed, as shown in Figure 3d–f. The diameter of core–shell nanocrystals increased to 40 nm and 50 nm with a 17 nm thickness. These group data suggest that core nanocrystals that are too small would inhibit the transversal shell growth. It is difficult to achieve ultrathin 2D NaYF_4_ by using a smaller core (e.g., less than 10 nm), because the thickness of core–shell nanocrystals would increase appropriately in the transversal shell growth. 

This can be explained by the thermodynamics of crystal growth. Thermodynamics tells us that the Gibbs free energy for a spherical particle can be expressed as the sum of the volume energy and the surface energy change (Equation (1)):

∆*G_tot_* = ∆*G_vol_* + ∆*G_sur_* = A*d^3^G*_v_* + B*d^2^*γ_u_* (1)

where ∆*G_tot_* is the total energy change from the initial state to final state, ∆*G_vol_* is the volume energy change, ∆*G_sur_* denotes the energy change on the surface, *d* is the size of the nanoparticle, *G_v_* is the energy per unit volume, A*d^3^ denotes the volume of the nanoparticle, B*d^2^ is the surface area of the nanoparticle, *A* and *B* are two constants, and *γ_u_* is the surface energy per unit area of the surface. For smaller nanoparticles, there is a larger fraction of their atoms on the surface. To reduce the value of ∆*G_sur_* and the surface area, the core nanocrystal will tend to grow isotropically into a spherical shape rather than other shapes. As the size of nanocrystals increases, the fraction of atoms on the surface decreases quickly, and the anisotropic crystallographic property of the core nanocrystal performs, which allows the core nanocrystal to grow transversally. 

**Figure 4:**
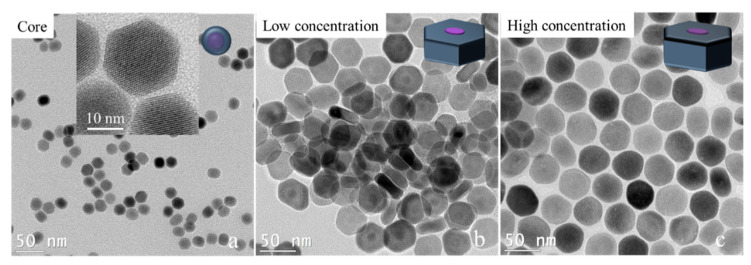
TEM images of NaGdF_4_/NaYF_4_ core nanocrystals before (**a**) and after shell growth with a low shell precursor concentration (**b**) and a high shell precursor concentration (**c**). Insert in (**a**) is the high-resolution TEM image for the corresponding sample.

We tried to break past the limit of synthesizing ultrathin 2D core–shell nanocrystals by adjusting the shell precursor concentration. As we know, besides the temperature, the concentration of shell precursors will affect the shell growth rate. Especially in the anisotropic shell growth process, a lower shell concentration or a slower shell precursor injection will slow the shell growth rate, which will promote the shell’s growth in the transversal direction. Here, we experimentally prove this hypothesis. To avoid the effect of small size on the transversal shell growth of nanocrystals, 16 nm × 21 nm NaGdF_4_/NaYF_4_ nanocrystals were prepared first. The NaGdF_4_/NaYF_4_ nanocrystals were used as cores for the transversal shell growth under a lower concentration and a higher concentration of the NaYF_4_ shell precursor while keeping the other conditions the same (see the experimental details in the Methods section). The TEM image results are shown in Figure 4. The low shell precursor concentration results were 13 nm × 28 nm NaGdF_4_/NaYF_4_ nanocrystals, while the high shell precursor concentration results were 28 nm × 41 nm NaGdF_4_/NaYF_4_ nanocrystals. The large difference in the thickness of the two disk-like nanocrystals demonstrates that the shell growth in the longitudinal direction was seriously inhibited. Even the thickness of core–shell nanocrystals was smaller than the core’s thickness, which suggests a negative growth occurred in the longitudinal direction (<001> orientation). This result inspires a new way to fabricate thinner two-dimensional core–shell nanocrystals by applying a low concentration of shell precursors in the condition of transversal shell growth.

**Figure 5:**
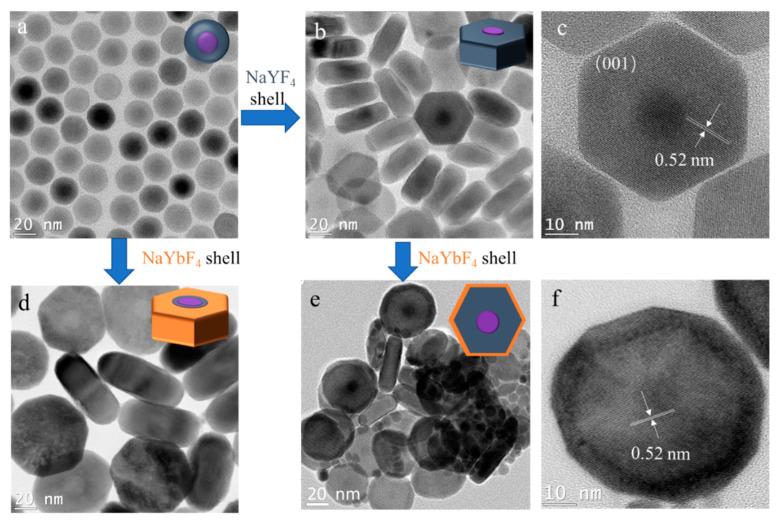
TEM images of NaGdF_4_/NaYF_4_ spherical nanocrystals as core (**a**) for transversal growth with different host shells: NaYF_4_ shell (**b**) and NaYbF_4_ shell (**d**). NaGdF_4_@NaYF_4_ nano-disk (**b**) as core for another transversal shell growth with a layer of NaYbF_4_ shell (**e**). High-resolution TEM images of NaGdF_4_/NaYF_4_ nano-disks (**c**) and NaGdF_4_/NaYF_4_/NaYbF_4_ nano-disks (**f**).

Next, we studied the effect of different shell hosts on the transversal shell growth. In order to easily characterize the core–shell structure, 25.5 nm NaGdF_4_/NaYF_4_ core–shell nanocrystals were used as the core (Figure 5a). The cores were transversally grown with NaYF_4_ shell and NaYbF_4_ shell, respectively, as shown in Figure 5b,d. The core–shell structure was clearly observed after the shell growth. The dark dots in the TEM images of NaGdF_4_/NaYF_4_ nanocrystals (Figure 5a–c) are NaGdF_4_, while the grey white dots in the TEM images of NaGdF_4_/NaYF_4_/NaYbF_4_ nanocrystals (Figure 5d) indicate the core of NaGdF_4_/NaYF_4_, because the Yb atom is heavier than the Gd and Y atoms, and the Gd atom is heavier than the Y atom. The similar disk-like shapes of the two obtained nanocrystals illustrates that the different shell hosts only had a minor influence on the transversal shell growth. Interestingly, the thickness of NaGdF_4_/NaYF_4_ nano-disk was 17.9 nm—smaller than the 25.5 nm size of the original core nanocrystal (Figure S3). The thickness of the nano-disk was further decreased to 14.3 nm after utilizing NaGdF_4_/NaYF_4_ nano-disks as cores for the growth of another layer of NaYbF_4_ shell (Figure 5e). The multiple-layer shell structure is clearly presented in the high-resolution TEM image (Figure 5f). The continuous reduction in the thickness of the nano-disk is coherent with the result obtained when applying the low shell precursor concentration (Figure 4b). This result further demonstrates the feasibility of synthesizing thinner 2D NaLnF_4_ nanocrystals via continuous transversal shell growth.

## 4. Conclusions

This work presented a highly controllable transversal shell growth method with a relatively lower reaction temperature compared to conventional methods. This method is advantageous to decrease the cation diffusion at the core–shell interface, which may promote the luminescence property of nanocrystals with separate doping area. Additionally, this method can increase or decrease the thickness of NaLnF_4_ nanocrystals, which could be useful for fabricating ultra-thin nanocrystals or multi-layer 2D nanocrystals. We conducted an in-depth study of the mechanism of the epitaxial shell growth of NaLnF_4_ nanocrystals, carefully discussed the major factors for controlling the shell growth direction and drew conclusions regarding the relationship between these factors, providing theoretical support for engineering the nanostructure of multifunctional heterogeneous nanocrystals. 

## Data Availability

Data is contained within the article or Supplementary Materials. Further, the published data can be reused by appropriate acknowledgement.

## Supplementary Materials

The following are available online at https://www.mdpi.com/2079-4991/11/3/654/s1, Figure S1: Extra TEM images and XRD patterns, Figure S2: XPS spectra, Figure S3: extra TEM images and size distributions.
