# Supplementary material for: Low-Temperature-Induced Controllable Transversal Shell Growth of NaLnF4 Nanocrystals
Source: Nanomaterials (Basel). 2021 Mar 8;11(3):654. doi: 10.3390/nano11030654 (PMC7999601; doi:10.3390/nano11030654)
Supplement: Supplementary file 1 [file nanomaterials-11-00654-s001.pdf]

nanomaterials

Supporting information

Low-Temperature-Induced Controllable Transversal Shell Growth of $NaLnF_4$ Nanocrystals

Deming Liu [1,2,*,†], Yan Jin [1,2,†], Xiaotong Dong [1,3], Lei Liu [1,2], Dayong Jin [4,*], John A. Capobianco [5,*] and Dezhen Shen [1,2,*]

1 State Key Laboratory of Luminescence and Applications, Changchun Institute of Optics Fine Mechanics and Physics Chinese Academy of Sciences, Changchun 130033, China; jinyan@mails.ucas.edu.cn (Y.J.); dpldyx@126.com (X.D.); liulei@ciomp.ac.cn (L.L.)
2 Center of Materials Science and Optoelectronics Engineering, University of Chinese Academy of Sciences, Beijing 100049, China
3 School of Chemistry and Chemical Engineering, Guizhou University, Guiyang 550025, China
4 Institute for Biomedical Materials and Devices, Faculty of Science, University of Technology Sydney, Sydney, NSW 2007, Australia
5 Department of Chemistry and Biochemistry, and Center for NanoScience Research, Concordia University, Montreal, QC H4B 1R6, Canada
* Correspondence: liudeming@ciomp.ac.cn (D.L.); dayong.jin@uts.edu.au (D.J.); john.capobianco@concordia.ca (J.A.C.); shendz@ciomp.ac.cn (D.S.)
† These authors contributed equally to this work.

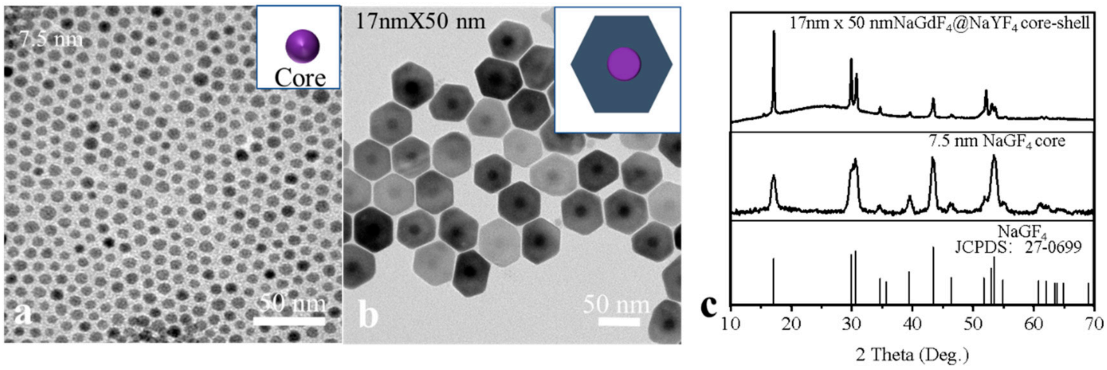

Figure S1. TEM images of 7.5 nm $NaGdF_4$ cores (**a**) and 17 × 50 nm $NaGdF_4@NaYF_4$ core-shell nanocrystals (**b**), scale bars are 50 nm. XRD diffractograms of 7.5nm β-$NaGdF_4$ cores and 17 × 50 nm β-$NaGdF_4@NaYF_4$ core-shell nanocrystals (**c**).

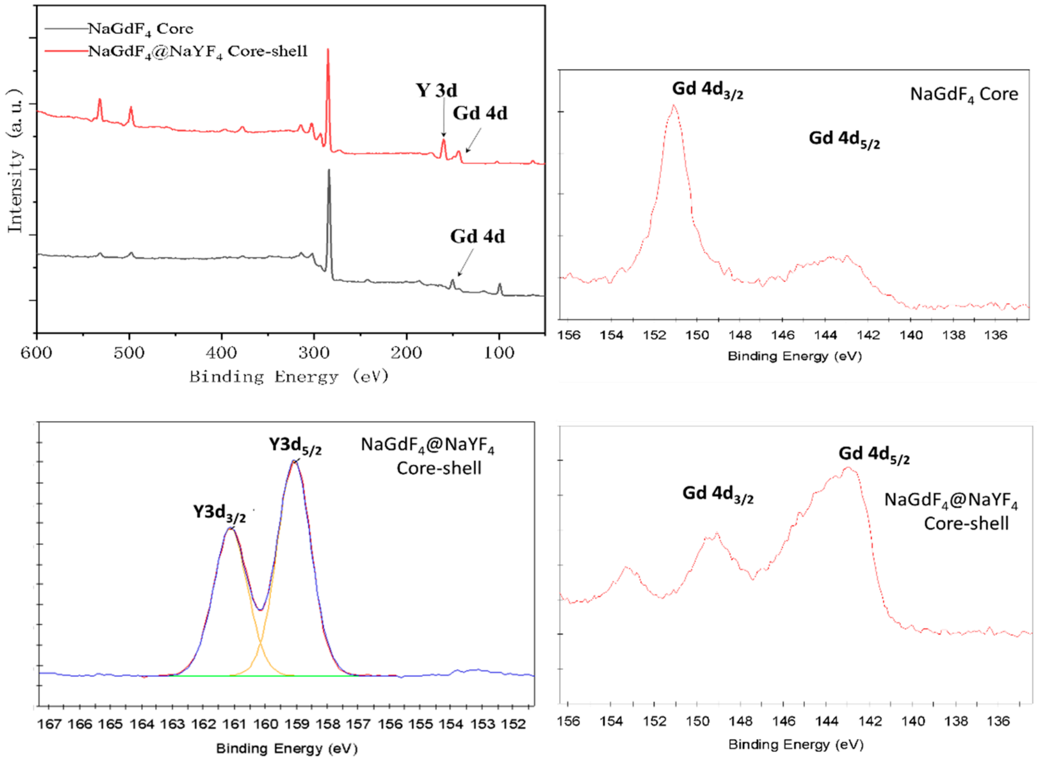

Figure S2. XPS spectra of 7.5 nm $NaGdF_4$ cores and 17 × 50 nm $NaGdF_4@NaYF_4$ core-shell nanocrystals.

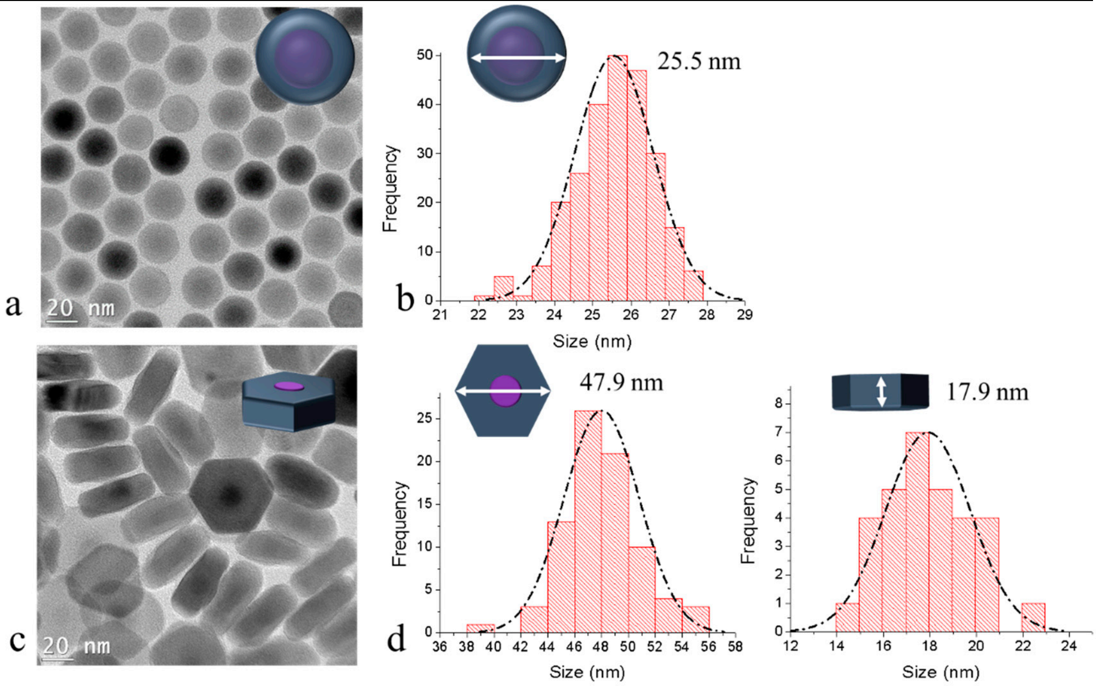

Figure S3. TEM images of $NaGdF_4@NaYF_4$ as cores before (**a**) and after (**c**) transversal shell growth with $NaYF_4$ shell and their size distributions (**b and d**). Scale bar is 20 nm.
